# Metabolic and Stress Responses in Senegalese Soles (*Solea senegalensis* Kaup) Fed Tryptophan Supplements: Effects of Concentration and Feeding Period

**DOI:** 10.3390/ani9060320

**Published:** 2019-06-05

**Authors:** Marcelino Herrera, Juan M. Miró, Inmaculada Giráldez, Natalia Salamanca, Juan A. Martos-Sitcha, Juan M. Mancera, Jose R. López

**Affiliations:** 1 IFAPA Centro Agua del Pino, km 4, Carretera El Rompido-Punta Umbría, 21450 Cartaya, Huelva, Spain; natalia.salamanca@juntadeandalucia.es (N.S.); lasacias@yahoo.es (J.R.L.); 2 Laboratorio de Biología Marina, Seville Aquarium R+D+I Biological Research Area, Zoology Department, University of Seville, Avd. Reina Mercedes 6, 41012 Seville, Spain; jmmiro@us.es; 3 Dpto. Química “Prof. J.C. Vilchez Martín”, Faculty of Experimental Sciences, Research Center in Technology of Products and Chemical Processes, PRO2TECS, University of Huelva, Campus de Excelencia Internacional del Mar (CEI·MAR), Avda. Fuerzas Armadas s/n, 21071 Huelva, Spain; giraldez@uhu.es; 4 Department of Biology, Faculty of Marine and Environmental Sciences, Instituto Universitario de Investigación Marina (INMAR), Universidad de Cádiz, Campus de Excelencia Internacional del Mar (CEI·MAR), 11519 Puerto Real, Cádiz, Spain; juanantonio.sitcha@uca.es (J.A.M.-S.); juanmiguel.mancera@uca.es (J.M.M.)

**Keywords:** feeding, metabolism, Senegalese sole, stress, welfare, tryptophan

## Abstract

**Simple Summary:**

In order to improve fish welfare in fish farms, feed additives (tryptophan, Trp) were assayed in a cultured species, the Senegalese sole (*Solea senegalensis*). It has been observed in previous studies that fish fed Trp supplements for several days prior to a stress challenge improved their physiological stress response. Therefore, this feeding strategy could be applied in commercial fish farms before submitting fish to stressful zootechnical procedures, such as grading, sampling, slaughter, and, consequently, fish welfare would be improved.

**Abstract:**

The objective of this study was to assess the impact of different dietary Trp concentrations on the stress and metabolism response of juvenile Senegalese soles (*Solea senegalensis*). Fish (38.1 ± 1.9 g) were fed different Trp-enriched feeds (0%, 1% and 2% Trp added) for two and eight days, and later exposed to air stress for three min. Samples were taken pre- and 1 h post-stress (condition). Plasma cortisol, lactate, glucose and proteins were significantly affected by the sampling time, showing higher values at 1 h post-stress. Trp concentration in food also had significant effects on lactate and glucose levels. However, the feeding period did not affect these parameters. Post-stress values were higher than in the pre-stress condition for every plasma parameter, except for lactate in two days and 1% Trp treatment. Nevertheless, cortisol, glucose and lactate did not vary significantly between pre- and post-stress samplings in fish fed the 1% Trp-enriched diet for two days. The lack of variability in cortisol response was also due to the high pre-stress value, significantly superior to pre-stress control. The exposure time to Trp feeding did not significantly affect any enzyme activity; however, Trp added and condition influenced protein-related enzyme activities. In spite of decreasing stress markers, Trp-enriched diets altered the protein metabolism.

## 1. Introduction

Effective fish farming requires good management, including a focus on maintaining fish welfare. It has been described that stressed farmed fish are prone to pathologies and present lower growth rates [1]. Moreover, stressful rearing procedures affect flesh quality; for instance, fish stressed before slaughter show lower muscle pH and faster meat quality deterioration [2,3]. Therefore, fish farmers try to attenuate fish stress by applying several techniques such as reduction of handling and lower stocking densities [1,4]. Some researchers have also focused on the use of feed additives to improve fish welfare, for instance amino acids (mainly tryptophan), vitamins, and probiotics [5,6,7,8]. Recent evidence also showed that stressful husbandry conditions affect amino acid metabolism in fish, and in some stress situations an increase in the requirement of certain essential amino acids may occur [9].

Tryptophan (Trp) is an essential amino acid that can only be acquired through diet since animals are not able to synthesize it endogenously. Trp is the serotonin (5-hydroxytryptamine, 5-HT) precursor, a monoamine which is simultaneously a neurotransmitter in the central nervous system and a paracrine or endocrine signal in the gut and blood [10]. In fish, 5-HT is involved in the hypothalamus-pituitary-interrenal (HPI) axis regulation, affecting the stress response. Over 95% of the ingested Trp is catabolized primarily in the liver via the kynurenine pathway and produces niacin, pyruvate and acetyl-CoA as the final products [11].

Tryptophan supplements in diet have been assayed in several animals, mainly mammals. In this regard, Le Floc’h and Seve [12] have reported different effects on stress response and immune systems in pigs; it has also been demonstrated that this amino acid can steady horses and dogs [13,14]. In fish, the physiological effects of dietary Trp supplementation have mainly been studied in freshwater species, such as tilapia (*Oreochromis niloticus* Linnaeus), rainbow trout (*Oncorhynchus mykiss* Walbaum), and white carp (*Cirrhinus mrigala* Hamilton) [15,16,17,18]. These studies suggest that Trp effects depend on the species since both positive (for instance reduction of post-stress plasma cortisol concentration and serotoninergic activity increased) and neutral effects on the stress response have been described.

However, most of the studies on dietary Trp in fish are based on the study of serotonin formation, largely due to its effect on stress markers. Stress is also related to metabolism (mainly energy metabolism), in which kynurenine is involved. Kynurenine metabolites, like pyruvate and acetyl-CoA, can enter the Krebs and glycolysis pathways to obtain energy [11]. Thus, the study of enzyme activity from intermediate metabolism would be interesting to examine if diet supplementation of Trp significantly affected metabolic pathways.

The Senegalese sole (*Solea senegalensis* Kaup) is a high market value species from the southern European coast and is cultured in several commercial fish farms, including in northern countries. Currently, this species is only cultured in Europe (Spain, Iceland, France, and Portugal), reaching 1600 MT in 2017, much less than other European flatfish species like the nearly 12,000 MT of turbot (*Scophthalmus maximus* Linneaus). Therefore, the studies focusing on the knowledge of its physiology and the improvement of its culture are still necessary and of great interest.

The endocrine, immune, and metabolic stress responses of farmed Senegalese soles (*Solea senegalensis* Kaup) have been reported under several stressors such as salinity, stocking density or temperature [19,20,21,22,23,24]. However, attenuating these responses through amino acid supplements has scarcely been studied. Only Costas et al. [23,25,26,27] compared the physiological stress responses in Senegalese soles fed experimental diets with different amino acid profiles (including Trp content), stating that several combinations enhance the lysozyme activity and decrease the plasma lactate and glucose concentrations. In addition, Azeredo et al. [28] studied the immunological effects of Trp additives in daily meals, showing that they enhanced the ACH50 (Alternative Complement Pathway) and disease resistance (assessed by bacterial challenge).

In addition to amino acid supplementations, the feeding period is another variable to be considered [23,29]. In other fish species, the concentration and feeding period with Trp-enriched feeds are variable. It would be expected that cold-water species, due to their slower metabolism and nutrient assimilation, need more time to incorporate these supplements [30,31]. Nevertheless, most species (including cold water species) can be affected by Trp additives after seven days and 1–2% Trp addition [16,17,32,33,34]. For instance, salmon (*Salmo salar* Linnaeus) fed Trp supplements for seven days reduced their plasma cortisol levels enhancing as a result of crowding and confinement stress [35,36]. Similarly, the plasma glucose and cortisol concentrations decrease in an added-Trp dependent manner in Atlantic cod (*Gadus morhua* Linnaeus) fed different Trp supplements for seven days and subjected to confinement stress [37]. However, Herrera et al. [34] reported that, despite the attenuating effects of these diets, the energy metabolism of fish can be negatively affected for feeding periods of seven days. It would be interesting to decrease the feeding period in order to prevent a metabolic imbalance due to an excess of dietary Trp.

The objective of this work is to study the short-term stress and metabolic responses in Senegalese soles fed different Trp concentrations, in order to determine the most suitable dosage and feeding time for minimizing metabolic alterations and attenuating stress.

## 2. Material and Methods

### 2.1. Experimental Feed and Analysis

Commercial fish feed (Le-2^®^ Skretting, Burgos, Spain) was used as the control diet. L-tryptophan (dry powder) was purchased from Jizhou Huaheng Biological Technology Co., Ltd. (Jizhou, China). The commercial control diet was finely ground and then mixed with water (400 mL kg^−1^ dry feed) and Trp supplementations. The amount of Trp varied in each of the experimental diets after adding 0% (control), 1% (1Trp), or 2% (2Trp) of Trp dry power (on dry feed weight) (Table 1). The mixture was thread pelleted into 2 mm diameter and 20–25 cm length strips. These food strips were dried at 60 °C for 24 h. Finally, these were cut into 2–3 mm size pellets, which were stored at 4 °C. Those values (1–2% Trp added) were chosen in order to obtain a final Trp concentration of two- and five-fold the control (Table 1).

As the Trp added could decrease through feed processing, all experimental diets were later analyzed through gas chromatography-mass spectrometry (GC-MS) to determine the final amino acid content. Feed homogenization was done through basic hydrolysis, as reported in Dai et al. [38]. For derivatization, an aliquot (100 µL) of standard solution or sample was placed in a 2 mL vial, adding 400 µL of a water:ethanol:pyridine (60:32:8) mixture and 40 µL of ethyl chloroformate. It was capped and vigorously shaken in a vortex mixer for 30 s at room temperature. Subsequently, 200 µL of chloroform (containing 1% elemental chlorine-free) was added and the derivatives were extracted into the organic phase by striking the tube against a pad for about 30 s. The organic phase was dried with anhydrous sodium sulphate. The organic layer was transferred into a new vial with a 300 μL fixed insert. Aliquots (1 μL) of the derivatized extracts were injected into a Shimadzu GC-MS (GCMS-TQ8030) equipped with an Agilent HP-5MS fused silica capillary column (30m × 0.25mm i.d., 0.25 mm film thickness). The gas chromatograph system was equipped with a split/splitless injection port operating in splitless mode. The oven temperature was programmed from 40 °C (5 min) to 270 °C (20 min) by increasing the temperature at 5 °C min^−1^. The transfer line was heated at 280 °C. The carrier gas was helium with a constant flow of 1 mL min^−1^ (mean velocity 36 cm s^−1^). The mass spectrometry was performed with electron ionization (EI) at 70 eV, operating in scan mode (75–500 amu). Identification of derivatized amino acids was achieved comparing the gas chromatograph retention times and mass spectra with those of the pure standard compounds. All mass spectra were also compared with the data system library (NIST 11). Quantification of samples was conducted by the external standard method following the same procedure as that for samples. Feed analysis results are shown in Table 1.

### 2.2. Experimental Desing

Senegalese sole (*Solea senegalensis*) juveniles came from natural spawns of the IFAPA Agua del Pino (Cartaya, Spain) broodstock. Experimental fish were kept in a marine recirculating system. The mean temperature, salinity, and dissolved oxygen were 19.7 ± 0.5 °C, 37 g L^−1^ and 6.5 ± 0.2 ppm (161 ± 3.3%), respectively. Photoperiod was 12L:12D. Under those rearing conditions, fish were acclimated to the experimental tanks 10 days prior to the experiment. The feeding for 10 days was ad libitum with commercial fish feed (Le-2^®^ Skretting), using 24 h clock-feeders. From day 11 to 50, the fish feed was switched to the control experimental feed (see below), and the ration adjusted to 1% tank biomass daily, checking the presence of remaining feed daily in order to be sure the feed was supplied in excess (this feed was removed and discarded). Fish weight was 38.1 ± 1.9 g (mean ± SE, N = 250). The experimental rearing was carried out in 10 PVC rectangular tanks (25 L, 55.5 cm × 35.5 cm × 13 cm), at a non-stressing stocking density for this species at this stage, 4.7 kg m^−2^ or 38 kg m^−3^ (25 fish tank^−1^) [20].

Several experimental feeds (duplicated) were applied following the feeding conditions described above, combining two factors: (1) Extra Trp amount added (0%—control, 1%—1Trp or 2%—2Trp); and (2) feeding period before subjecting to stress (two or eight continued days). The control group was fed the same fish feed with no Trp addition. Therefore, the experiment consisted of 4 treatments plus control (control, 1Trp2, 1Trp8, 2Trp2, and 2Trp8), and later sampled pre- and 1 h post-stress (condition factor). Trp amounts and feeding times were selected according to previous studies on other fish species [16,17,32,33,34], adjusting them to their possible minimal values (see Introduction).

The experiment complied with the Guidelines of the European Union Council (2010/63/EU) and the Spanish Government (RD1201/2005; RD53/2013 and law 32/2007) for the use of laboratory animals. According to RD1201/2005, M. Herrera is certified (type C) for working with and designing experiments with animals, and the aquaculture technicians involved in this study have the A-type Certificate, required for laboratory animal maintenance and care. All experimental protocols were approved by the Ethical Committee of the IFAPA (Andalusian Institute of Agricultural and Fisheries Research and Training), located in Seville (Spain).

### 2.3. Sampling

There were two samplings for every treatment (feeding type × feeding duration) under two different conditions: pre- and post-stress. Pre-stress samples were taken from fish (*n* = 10) without submitting to stress before directly catching them from the tanks. For post-stress samples, fish were exposed to air for three min just after collecting, which is a common procedure used in stress research in fish since it leads to an acute stress response [25,34,39,40]. Therefore, all fish were netted and kept out of the water for three minutes; subsequently, they were taken back to the tanks and sampled (*n* = 10) at one-hour post-stress (hps) since plasma stress parameters (i.e., cortisol and lactate) show their peaks around that time [41]. In summary, 10 pre- and 10 post-stress samples were obtained for every treatment: control, 1Trp2, 1Trp8, 2Trp2, and 2Trp8; therefore, the total number of samples was 20 × 5 = 100 (from every tissue/plasma).

Fish were sacrificed through anesthetics overdose (immersion in >1 mL L^−1^ 2-phenoxyethanol) and blood and liver were sampled. Blood (500–600 µL) was collected by puncture from the caudal peduncle into 1 mL heparinized syringes (25,000 units of ammonium heparin/3 mL saline solution 0.6% NaCl, Sigma H6279). Plasma was separated from cells by centrifugation of the whole blood (3 min, 10,000× *g*, 4 °C), snap frozen in liquid nitrogen and stored at −80 °C until analysis of glucose, lactate, proteins, and cortisol. The liver was removed from each fish, frozen in liquid nitrogen, and stored at −80 °C until assayed.

### 2.4. Plasma and Liver Analyses

Plasma glucose, proteins, and lactate levels were measured using commercial kits from Química Analitica Aplicada S.A. (QCA Glucose Liquid Ref. 998225, QCA Total Proteins Ref. 997180, Tarragona, Spain) and Spinreact (Lactate Ref. 1001330, Barcelona, Spain) adapted to 96-well microplates [24]. All assays were performed with a Tecan Sunrise microplate reader, using Magellan v2.5 software for Windows (Tecan Austria, Salzburg, Austria).

Plasma cortisol levels were quantified by an ELISA kit (EA65, Oxford Biomedical Research, Rochester Hills, MI, USA) modified and adapted to fish [42]. Cortisol was extracted from 20 μL plasma in 200 µL diethyl ether. The lower limit of detection (81% of binding) was 0.1 ng mL^−1^ plasma. The inter-assay coefficient of variation was 9.8%, while the mean intra-assay coefficient of variation was 4.6%. The mean percentage of recovery was 90%. The main cross reactivities (>1%; given by the supplier) were detected with corticosterone (3.38%), cortisone (2.08%), and deoxycorticosterone (2%).

Livers were homogenized by ultrasonic disruption with 10 volumes of ice-cold stopping-buffer containing 50 mmol L^−1^ HCl (pH 7.5), 15 mmol L^−1^ 2-mercaptoethanol, 100 mmol L^−1^ KF, 5 mmol L^−1^ EDTA, 5 mmol L^−1^ EGTA, and a protease inhibitor cocktail (Sigma, P-2714). The homogenate was centrifuged (30 min at 10,000× *g*, 4 °C, Eppendorf 5415R) and the supernatant was used in enzyme assays and protein analyses.

Reaction rates of enzymes were determined by changes in absorbance of NAD(P)H at 340 nm using the Tecan Sunrise microplate reader. The reactions were started by addition of homogenates (15 μL) at a pre-established protein concentration, omitting the substrate in control wells (final volume of 275–295 μL) and allowing the reactions to proceed at 25 °C for pre-established times (5–15 min). Protein levels were assayed in duplicate with the QCA Total Proteins kit (Química Clínica Aplicada S.A., Barcelona, Spain). Enzymatic analyses were carried out at conditions meeting requirements for optimal velocities. Enzyme activities involved in amino acid catabolism, gluconeogenesis and glycolysis were assessed: alanine transaminase (AlaT; EC 2.6.1.2), aspartate transaminase (AspA; EC 2.6.1.1), glutamate dehydrogenase (GDH; EC 1.4.1.2), fructose biphosphatase (FBP; EC 3.1.3.11), glucose 6-phosphate dehydrogenase (G6PDH; EC 1.1.1.49), phosphofructokinase (PFK; EC 2.7.1.11) and pyruvate kinase (PK; EC 2.7.1.40). The specific conditions for the assay of those enzymes are described in the literature [43,44,45,46,47]. The activities were expressed as mU/mg protein.

### 2.5. Statistical Analysis

Normality and homoscedasticity of all data sets were checked through the Kolmogorov-Smirnov and Levene tests, respectively (SPSS v.21.0, IBM, Armonk, NY, USA). A nested Analysis of Variance (ANOVA) was performed to check the tank (replicates) effects on every variable. To study the plasma parameters and enzyme activities variation according to treatments, a three-way ANOVA was performed with the three factors: feeding time (2 levels: 2 and 8 days), Trp added (3 levels: 0%, 1%, and 2%) and condition (2 levels: pre-stress and 1 hps). The general effects of every variable and their interactions were analysed. Later post-hoc analyses were performed to detect significant differences between groups. For that purpose, Student-t tests were used in two level factors (feeding time and condition), and Bonferroni test for multiple comparisons from the Trp added factor. Data are expressed as mean ± standard error of mean (SE), and sample size was 10 for every treatment and variable. The significance level was 0.05.

## 3. Results

No fish mortalities were registered during the experimental period. No differences between replicated tanks were detected within every variable.

According to the three-way ANOVA, plasma cortisol, lactate, glucose and proteins were significantly affected by different factors (Table 2), usually showing higher values in the post-stress sampling. The interaction of the three factors was significant for plasma cortisol; therefore, the effects on condition (significant statistically) are influenced by the other two factors. For glucose, every pair of interactions was significant while the triple interaction was not. Only Trp*CD interaction was significant for plasma lactate, indicating that FP does not influence these metabolite variations. Plasma proteins were only affected by the condition (pre-stress values lower than post-stress ones).

**Figure 1:**
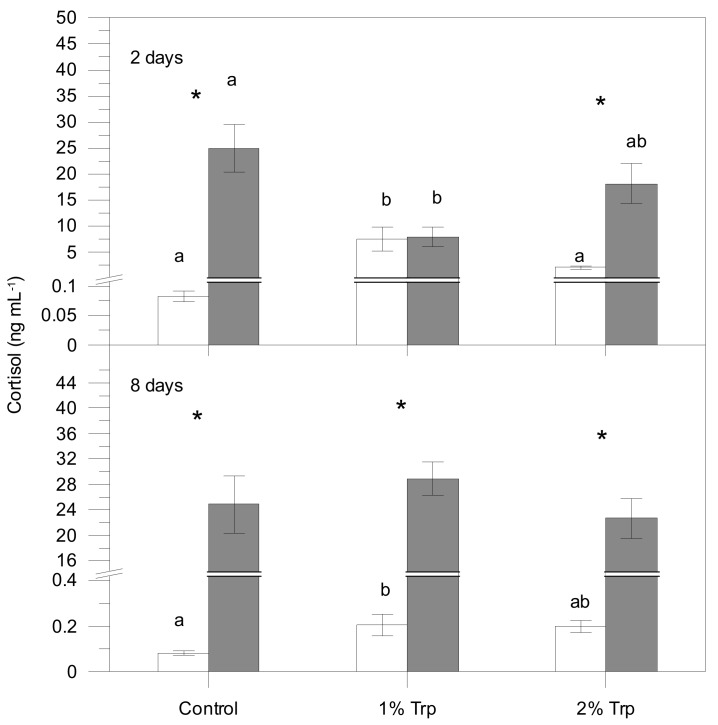
Plasma cortisol concentration for each treatment (mean ± SE). White and grey bars are pre- and post-stress values, respectively. Asterisks indicate significant differences between pre- and post-stress conditions. Different letters indicate significant differences among groups (different experimental feeds) within each sampling (pre- and post-stress).

**Figure 2:**
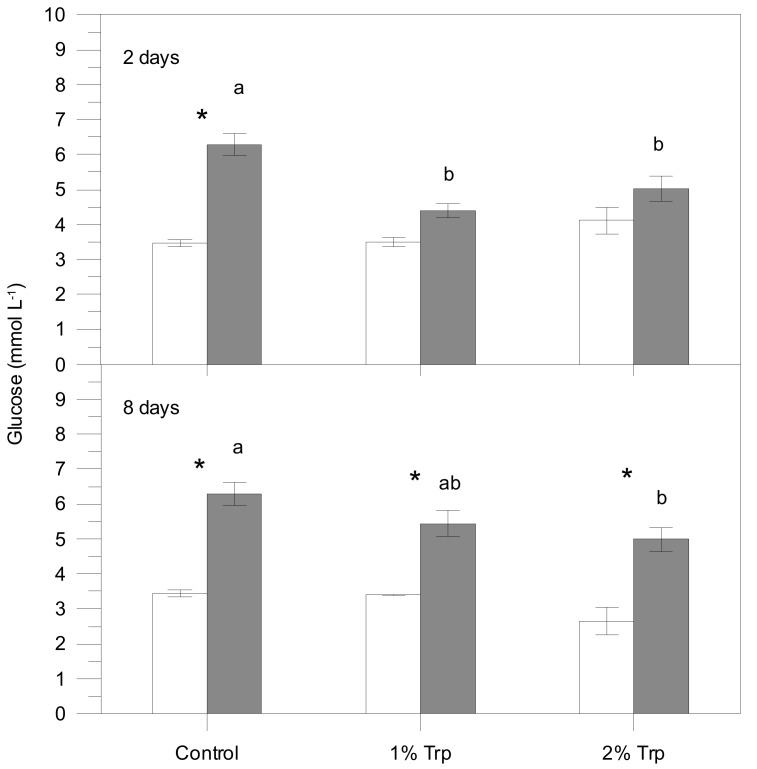
Plasma glucose concentration for each treatment (mean ± SE). White and grey bars are pre- and post-stress values, respectively. White and grey bars are pre- and post-stress values, respectively. Asterisks indicate significant differences between pre- and post-stress conditions. Different letters indicate significant differences among groups (different experimental feeds) within each sample (pre- and post-stress).

**Figure 3:**
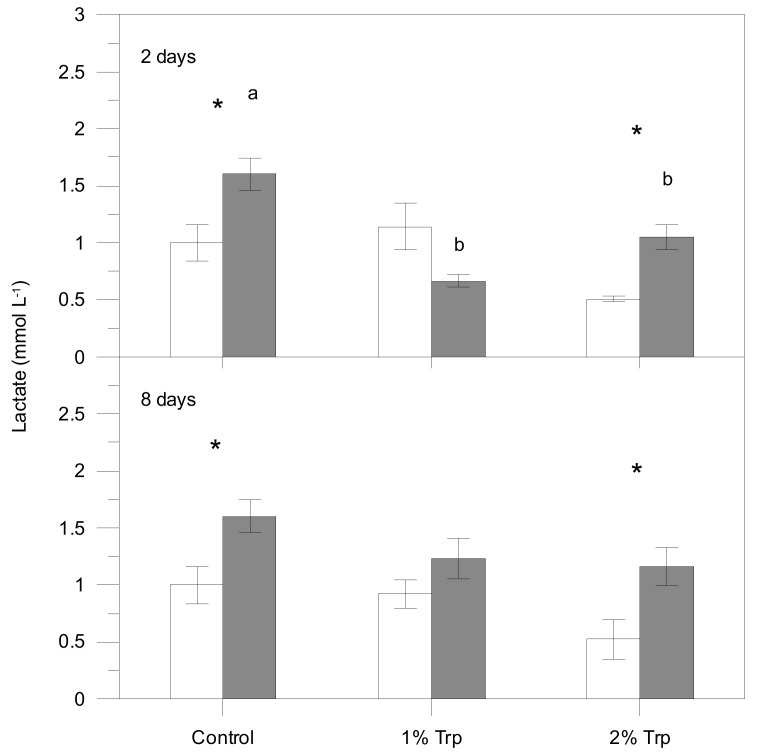
Plasma lactate concentration for each treatment (mean ± SE). White and grey bars are pre- and post-stress values, respectively. White and grey bars are pre- and post-stress values, respectively. Asterisks indicate significant differences between pre- and post-stress conditions. Different letters indicate significant differences among groups (different experimental feeds) within each sample (pre- and post-stress).

**Figure 4:**
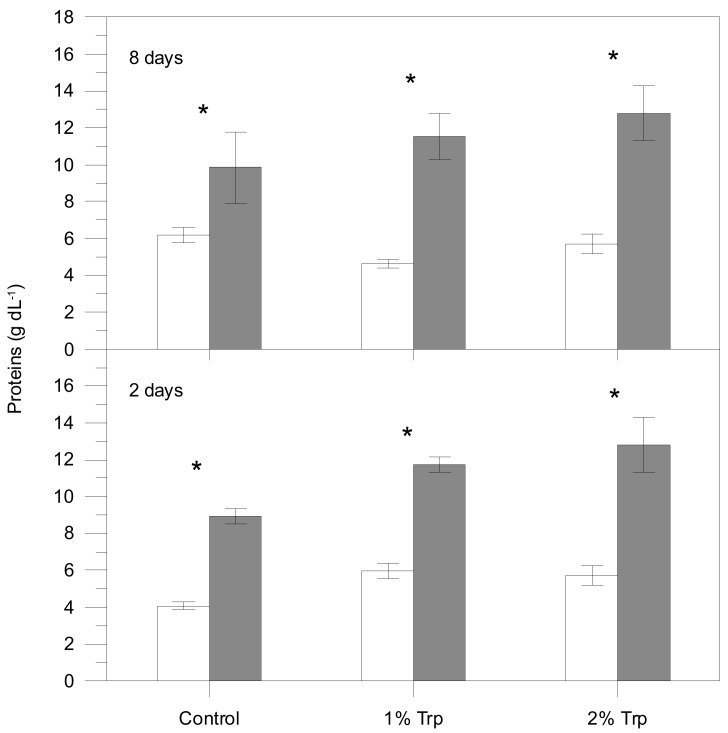
Plasma proteins concentration for each treatment (mean ± SE). White and grey bars are pre- and post-stress values, respectively. Asterisks indicate significant differences between pre- and post-stress conditions. Different letters indicate significant differences among groups (different experimental feeds) within each sample (pre- and post-stress).

Post-stress values for every Trp/FP group were generally higher than pre-stress values (Figure 1, Figure 2, Figure 3 and Figure 4). Those differences were significant for the control group in all plasma parameters. Nevertheless, cortisol, glucose and lactate did not vary significantly between pre- and 1 h post-stress conditions in the 1Trp2 treatment. There was a lack of variability in cortisol response to experimental feeds, although this hormone concentration was higher in stressed fish than pre-stress ones within the control feed. The addition of 1% Trp for 2 days reduced the post-stress glucose and lactate values significantly. Specifically only plasma lactate in 1Trp2 treatment decreased after submitting to air stress, though not significantly.

Overall, plasma parameters (except proteins) showed the least variation in the 1Trp2 treatment. Therefore, the minimum and maximum cortisol differences (stressed values minus non-stressed ones) were for the 1Trp2 and Control treatments, 0.40 and 24.76 ng mL^−1^, respectively. The same pattern was obtained for glucose—16.04 and 50.94 mg dL^−1^. The clear variations in the 8-day treatments were not very different from the control for every variable; only 1Trp8 lactate did not change significantly.

**Figure 5:**
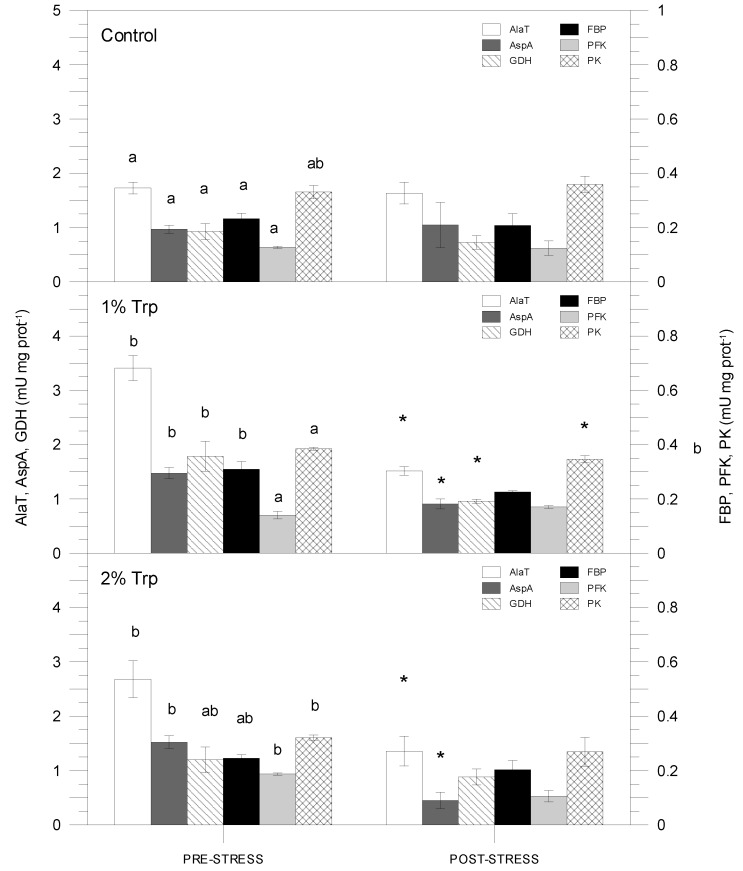
Hepatic enzyme activities (AlaT, AspA, GDH, FBP, PFK and PK) for the two-day treatment. Different letters indicate significant differences among groups (experimental feeds) within each condition, and asterisks show significant differences between pre- and post-stress condition within each feed type.

**Figure 6:**
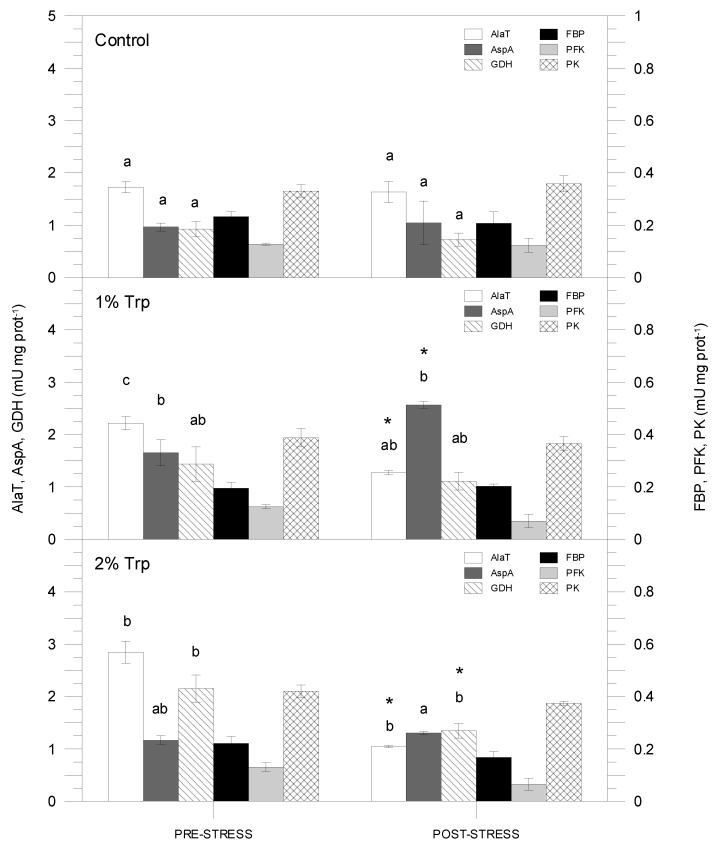
Hepatic enzyme activities (AlaT, AspA, GDH, FBP, PFK and PK) for the eight-day treatment. Different letters indicate significant differences among groups (experimental feeds) within each condition, and asterisks show significant differences between pre- and post-stress condition within each feed type.

Table 3 shows the three-way ANOVA results for enzyme activities. Only FBP and PFK did not show any significant variation. Figure 5 and Figure 6 show the enzyme activity variations among treatments. These activities did not vary after air exposure in the control group. G6PDH activity was not significantly different among any treatment (data not shown). The activities related to amino acid metabolism (AlaT, AspA and GDH) changed significantly between the non-stressed and stressed condition, and moreover, significant differences were detected in the control. The highest enzyme activities were registered for the AlaT, showing the maximum in the pre-stress 1Trp2 treatment (3.41 mU mg prot^−1^). Finally, the PFK showed the lowest enzyme activities, the minimum being for the post-stress 2Trp8, 0.17 mU mg prot^−1^.

## 4. Discussion

The role of dietary Trp supplements on stress attenuation has already been studied in several fish species, and both positive and no effects have been reported [17,18,23,32,48]. Nevertheless, this is the first study on the effects of Trp concentration, feeding duration and their interaction on the stress and metabolic responses in a flatfish, the Senegalese sole (*Solea senegalensis*). Overall, the results showed that Trp supplements affect the stress system, modulating significantly both plasma biomarkers and hepatic enzyme activities.

It is known that plasma cortisol rises after subjecting to stress [49]. This is in agreement with our 3-way ANOVA, where differences between pre- and post-stress condition were significant. However, the 1Trp2 treatment did not show those differences, although pre-stress cortisol levels were high. In mammals, it has been reported that treatments with serotonin precursors, such as Trp, stimulate the HPA (hypothalamus-pituitary-adrenal) axis reactivity, elevating plasma levels of glucocorticoids [50]. This is because serotonin terminals synaptically contact with CRH (Corticotropin Releasing Hormone) immunoreactive neurons within the hypothalamic paraventricular nucleus [51]. Lepage et al. [15] also described a pre-stress cortisol increase in rainbow trout fed Trp additives. Consequently, the authors have suggested that the observation that supplementary dietary Trp attenuated the stress-induced elevation of plasma cortisol may seem contradictory. However, they explained that this effect could be due to a negative feedback as a result of increased pre-stress plasma cortisol. Our results are in accordance with that statement and, in addition, demonstrate that the effects of Trp additives on cortisol changes are detectable in a short-term window (see below).

In this way, Basic et al. [35,37] also described suppressive effects of Trp supplements on post-stress cortisol after a short feeding time (1 day) as well as a lower attenuation of that hormone for longer exposure time. The authors suggested that that pattern reflects a rather short-term window for the effects of dietary Trp treatment on HPI-axis activity. Therefore, the cortisol production and releasing in groups feeding Trp-enriched diets could be inhibited in long-term assays, as in our eight-day treatments. Besides the high Trp concentration, the longer feeding time in those treatments could have induced a cortisol over-synthesis, which would have inhibited cortisol release in plasma.

However, Martins et al. [18] did not report plasma cortisol variations after stress in Nile tilapia (*Oreochromis niloticus*) fed dietary Trp supplements (1.87 and 4.45 % for 7 days). They stated that the use of very high Trp concentrations could reverse the effects observed with lower supplementation levels [52]. This is in agreement with our results since 2% Trp treatments did not attenuate the stress marker variations significantly except for the lactate. In addition, Wolkers et al. [53] reported that Trp-enriched diets do not always affect the HPI reactivity in the matrinxã (*Brycon amazonicus* Spix & Agassiz). Contrarily, Tejpal et al. [16] described a post-stress plasma cortisol linear decrease inversely related to the dietary Trp added in *Cirrhinus mrigala* Hamilton. Similar results have been reported by Kumar et al. [33] for rohu (*Labeo rohita* Hamilton). Both papers described long-term experiments (45 and 60 days, respectively). Nevertheless, that pattern was not detected in the present study, and the difference could be due to the feeding time (2 and 8 days) as well as the stressor and stress type (chronic stress in those papers versus acute stress in ours).

The stress secondary response markers (glucose and lactate) were significantly affected by the dietary Trp in terms of Trp concentration, although the feeding time did not seem to influence those parameters. In fact, the variations in eight-day treatments were very similar to those for the control.

In the control group, plasma glucose increased after air stress, similarly to what was reported previously for this species subjected to similar challenges [41,54]. Both 1% and 2% Trp supplementations attenuated the plasma glucose increase, and no significant differences were registered between pre-stress and stressed condition in the two-day experimental treatments. Similar results have been reported for other teleosts, including the Senegalese sole, during longer feeding time periods [16,23,33]. Maybe the longer feeding time promoted a metabolic adaptation to the experimental diets and circulating glucose returned to its normal values.

Plasma lactate did not change significantly in the 1% Trp treatments. To date, only Herrera et al. [34] have studied the post-stress lactate variations related to dietary Trp additives in the cod (*Gadus morhua* Linnaeus) after a short feeding period, despite it being a classical stress marker. The authors did not find significant differences among Trp-enriched and control groups, which could be due to the longer feeding period (seven days), thus supporting the hypothesis on the short efficiency time-window for Trp treatments (see above). According to our results, the 1Trp2 feeding did not lead to significant variations in both primary (cortisol) and secondary (glucose and lactate) stress markers [1,49].

Despite the high amino acid concentration in feed, plasma proteins were only affected by the condition, presenting higher values for the post-stress samplings. It is known that in marine teleost, acute stress due to plasma catecholamines rise altered gill permeability, inducing water loss and concomitantly plasma osmolality and protein concentration enhancement [49]. Our results agree with this pattern of change and revealed that Trp supplementation did not affect this increase.

Considering the classical plasma stress markers (cortisol, glucose and lactate), the 1Trp2 treatment showed the best results regarding stress attenuation, since no parameter changed significantly after air exposure stress in that treatment. Nevertheless, this treatment evoked a pre-stress cortisol increase; therefore, fish were slightly stressed before the air stress challenge because of the Trp-enriched diet.

With regard to the effects on energy metabolism, supplemented Trp influenced the metabolic enzymes in the pre-stress condition. Those differences were important mainly for enzymes related to amino acid metabolism, AlaT, AspA and GDH. It seems that dietary Trp enhanced those pathways. Although it has been described that stress enhances metabolism and the consumption of energy substrates [55], our results show that higher protein-related enzyme activities were registered in the pre-stress condition. This is in agreement with Hoseini et al. [17], who described decreased AlaT and AspA activities during metal exposure stress in carps (*Cyprinus carpio*) fed a Trp-enriched diet.

AlaT catalyzes the transfer of an amino group from L-alanine to α-ketoglutarate, the products of this reversible transamination reaction being pyruvate and L-glutamate. Alanine is an intermediary metabolite of Trp metabolism [56], and it could be the reason why AlaT activity was higher in Trp-enriched groups. AspA catalyzes the interconversion of aspartate and α-ketoglutarate to oxaloacetate and glutamate, although it also acts on tryptophan. Therefore, higher contents of Trp could have resulted in high AspA and AlaT activities. Coupled with AlaT, GDH is involved in the reaction of transdeamination, playing a central role in the metabolism of amino acids because it provides the principal route by which nitrogen is excreted from amino acids [57]. Therefore, their measurement can be a remarkable indicator of the metabolic utilization of excess amino acids by the fish [58,59,60], as suspected in our study. Nevertheless, those amino acid metabolism enzymes were less active in the pre-stress condition. It is known that stress is an energy-demanding process and energy reserves are used to cope with it [52]. In our experiments, the fastest and easiest ways to obtain energy were the amino acid pathways due to their elevated availability. This most likely occurred just after the air stress challenge and those amino acid reserves were quickly consumed; therefore, those enzyme activities were lower at 1 h post-stress. The significant alteration of the protein metabolism due to Trp additives has also been described by Herrera et al. [34], who suggested the use of another amino acid (phenylalanine) for attenuating stress responses since it did not affect the energy metabolism in cod.

G6PDH, PK and PFK are involved in the glycolysis and FBP is a key gluconeogenic enzyme. Overall these enzyme activities did not show significant changes; consequently, the carbohydrate pathways were not key in the stress response for Trp-enriched diets. In the Atlantic cod, a different pattern has been reported (Herrera et al., 2017). The authors described a general increase of those enzyme activities in Trp control treatments just after being subjected to stress. Therefore, they stated that that Trp-enriched diets negatively affect stress attenuation, keeping high gluconeogenic and glycolytic activities. Nevertheless, metabolizing amino acid substrates (in excess, see above) was the main metabolic strategy to cope with stress in the Senegalese sole. The differences in inherent physiology in each species could be the reason for different metabolic responses: cold/warm-water species; high/low metabolic rate; round/flat fish, among others.

## 5. Conclusions

Trp-enriched diets altered amino acid metabolism although they attenuated the stress response (assessed by classical plasma biomarkers as cortisol, glucose and lactate) in farmed Senegalese soles. The best results in terms of stress reduction were registered for the 1% Trp and 2-day treatment. From an aquaculture perspective, the use of a feed based on this Trp concentration for two days prior to a stress situation (inter alia transport, grading, sampling,) could improve fish welfare in fish farms, leading to an improved physiological status. Despite decreasing stress markers, protein metabolism was altered significantly through this diet; therefore, it is necessary to study alternative amino acid-based additives in order to reduce them.

## Figures and Tables

**Table 1:** Amino acid composition (g amino acid/kg food) for every experimental fish feed (concentration values are mean ± SE).

Amino Acid	Control (No Trp Added)	1Trp (1% Trp Added)	2Trp (2% Trp Added)
Glycine	3.6 ± 0.4	3.6 ± 0.4	3.6 ± 0.4
Valine	3.8 ± 0.5	3.8 ± 0.5	3.8 ± 0.5
Glutamic acid	64.7 ± 11.6	64.7 ± 11.6	64.7 ± 11.6
Leucine	7.3 ± 0.9	7.3 ± 0.9	7.3 ± 0.9
Threonine	0.3 ± 0.0	0.3 ± 0.0	0.3 ± 0.0
Isoleucine	1.1 ± 0.1	1.1 ± 0.1	1.1 ± 0.1
Proline	7.2 ± 0.8	7.2 ± 0.8	7.2 ± 0.8
Aspartic acid	48.1 ± 7.8	48.1 ± 7.8	48.1 ± 7.8
Methionine	1.9 ± 0.3	1.9 ± 0.3	1.9 ± 0.3
Phenylalanine	4.3 ± 0.6	4.3 ± 0.6	4.3 ± 0.6
Cysteine	0.1 ± 0.0	0.1 ± 0.0	0.1 ± 0.0
Lysine	15.9 ± 2.1	15.9 ± 2.1	15.9 ± 2.1
Histidine	1.1 ± 0.2	1.1 ± 0.2	1.1 ± 0.2
Tyrosine	5.8 ± 3.3	5.8 ± 3.3	5.8 ± 3.3
Glycine	3.6 ± 0.4	3.6 ± 0.4	3.6 ± 0.4
Tryptophan	2.5 ± 0.2	5.8 ± 0.3	12.5 ± 0.7

**Table 2:** Three-way ANOVA parameters (α = 0.05) for plasma cortisol, glucose, lactate, and proteins. Factors are feeding time (2/8 days), Trp added (control/1%/2%) and condition (pre-/post-stress); ns = no significant. F is in bold when effects are significant.

Substance		Feeding Period (FP)	Trp Added (Trp)	Condition (CD)	FP*Trp	FP * CD	Trp * CD	FP * Trp * CD
Cortisol	F	2.465	0.372	**124.3**	1.505	**10.77**	**2.977**	**6.147**
	*p*	ns	ns	<0.0005	ns	0.001	0.049	0.003
Glucose	F	0.216	**6.010**	**98.23**	**3.129**	**4.658**	**5.165**	1.425
	*p*	ns	0.003	<0.0005	0.048	0.033	0.007	ns
Lactate	F	0.356	**9.151**	**13.32**	0.111	3.313	**7.014**	2.497
	*p*	ns	0.0002	0.0004	ns	ns	0.001	ns
Proteins	F	3.255	0.17	**94.58**	0.963	1.597	0.0005	0.279
	*p*	ns	ns	<0.0005	ns	ns	ns	ns

**Table 3:** Three-way ANOVA parameters (α = 0.05) for enzyme activities. Factors are feeding time (2/8 days treatment), Trp added (control/1%/2%) and condition (pre-/post-stress); ns = no significant. F is in bold when effects are significant.

Enzyme		Feeding Period (FP)	Trp Added (Trp)	Condition (CD)	FP * Trp	FP * CD	Trp * CD	FP * Trp * CD
AlaT	F	1.058	**5.232**	**54.149**	1.180	3.160	**16.202**	1.161
	*p*	ns	0.010	0.000	ns	ns	0.000	ns
AspA	F	3.102	**5.881**	0.193	**2.778**	2.999	0.053	2.083
	*p*	ns	0.006	ns	0.041	ns	ns	ns
GDH	F	2.154	**13.792**	**22.623**	**2.761**	2.415	**3.484**	1.956
	*p*	ns	0.000	0.000	0.042	ns	0.041	ns
G6PDH	F	2.927	2.751	1.521	0.886	0.686	**5.976**	0.814
	*p*	ns	ns	ns	ns	ns	0.006	ns
FBP	F	0.273	0.321	1.542	0.206	0.117	0.608	0.117
	*p*	ns	ns	ns	ns	ns	ns	ns
PFK	F	1.903	0.017	0.776	0.985	0.920	0.156	2.409
	*p*	ns	ns	ns	ns	ns	ns	ns
PK	F	2.655	0.398	4.044	**2.926**	0.215	1.657	0.556
	*p*	ns	ns	ns	0.034	ns	ns	ns
